# Supplementary material for: Genome-Wide Association Study for Spot Blotch Resistance in Synthetic Hexaploid Wheat
Source: Genes (Basel). 2022 Aug 4;13(8):1387. doi: 10.3390/genes13081387 (PMC9407756; doi:10.3390/genes13081387)
Supplement: Supplementary file 1 [file genes-13-01387-s001.zip › Supplementary Table S1.pdf]

Table S1. Seedling spot blotch (SB) reaction scores of synthetic hexaploid wheat (SHW) lines derived from crosses between durum wheat (DW, *T. turgidum* L.) and *Aegilops tauschii* Coss (*Ae. squarrosa*) parents

		**Reaction to SB**		
Entry No.	**Pedigree**	**AVG****	**Score**	**Number of Entries**
1	**68.111/RGB-U//WARD***	3.6	S	7
2	68.111/RGB-U//WARD/3/AE.SQUARROSA (316)	1.3	R	
3	68.111/RGB-U//WARD/3/AE.SQUARROSA (321)	1.1	R	
4	68.111/RGB-U//WARD/3/AE.SQUARROSA (322)	1.6	MR	
5	68.111/RGB-U//WARD/3/AE.SQUARROSA (329)	2.2	MR	
6	68.111/RGB-U//WARD/3/AE.SQUARROSA (511)	1.6	MR	
7	68.111/RGB-U//WARD/3/AE.SQUARROSA (426)	2.2	MR	
8	68.111/RGB-U//WARD/3/AE.SQUARROSA (202)	1.9	MR	
9	**68.111/RGB-U//WARD RESEL/3/STIL***	2.7	MS	31
10	68.111/RGB-U//WARD RESEL/3/STIL/4/AE.SQUARROSA (164)	1.5	R	
11	68.111/RGB-U//WARD RESEL/3/STIL/4/AE.SQUARROSA (332)	1.1	R	
12	68.111/RGB-U//WARD RESEL/3/STIL/4/AE.SQUARROSA (781)	1.4	R	
13	68.111/RGB-U//WARD RESEL/3/STIL/4/AE.SQUARROSA (783)	1.5	R	
14	68.111/RGB-U//WARD RESEL/3/STIL/4/AE.SQUARROSA (385)	2.1	MR	
15	68.111/RGB-U//WARD RESEL/3/STIL/4/AE.SQUARROSA (386)	1.7	MR	
16	68.111/RGB-U//WARD RESEL/3/STIL/4/AE.SQUARROSA (389)	1.7	MR	
17	68.111/RGB-U//WARD RESEL/3/STIL/4/AE.SQUARROSA (390)	1.3	R	
18	68.111/RGB-U//WARD RESEL/3/STIL/4/AE.SQUARROSA (392)	1.0	R	
19	68.111/RGB-U//WARD RESEL/3/STIL/4/AE.SQUARROSA (1029)	1.7	MR	
20	68.111/RGB-U//WARD RESEL/3/STIL/4/AE.SQUARROSA (1030)	2.2	MR	
21	68.111/RGB-U//WARD RESEL/3/STIL/4/AE.SQUARROSA (1038)	1.6	MR	
22	68.111/RGB-U//WARD RESEL/3/STIL/4/AE.SQUARROSA (631)	1.7	MR	
23	68.111/RGB-U//WARD RESEL/3/STIL/4/AE.SQUARROSA (662)	2.0	MR	
24	68.111/RGB-U//WARD RESEL/3/STIL/4/AE.SQUARROSA (672)	1.5	R	
25	68.111/RGB-U//WARD RESEL/3/STIL/4/AE.SQUARROSA (685)	2.1	MR	
26	68.111/RGB-U//WARD RESEL/3/STIL/4/AE.SQUARROSA (623)	1.5	R	
27	68.111/RGB-U//WARD RESEL/3/STIL/4/AE.SQUARROSA (625)	1.7	MR	
28	68.111/RGB-U//WARD RESEL/3/STIL/4/AE.SQUARROSA (627)	1.4	R	

29	68.111/RGB-U//WARD RESEL/3/STIL/4/AE.SQUARROSA (628)	1.2	R	
30	68.111/RGB-U//WARD RESEL/3/STIL/4/AE.SQUARROSA (630)	1.2	R	
31	68.111/RGB-U//WARD RESEL/3/STIL/4/AE.SQUARROSA (631)	1.3	R	
32	68.111/RGB-U//WARD RESEL/3/STIL/4/AE.SQUARROSA (1090)	1.3	R	
33	68.111/RGB-U//WARD RESEL/3/STIL/4/AE.SQUARROSA (188)	1.3	R	
34	68.111/RGB-U//WARD RESEL/3/STIL/4/AE.SQUARROSA (659)	1.8	MR	
35	68.111/RGB-U//WARD RESEL/3/STIL/4/AE.SQUARROSA (675)	1.1	R	
36	68.111/RGB-U//WARD RESEL/3/STIL/4/AE.SQUARROSA (681)	1.8	MR	
37	68.111/RGB-U//WARD RESEL/3/STIL/4/AE.SQUARROSA (684)	1.2	R	
38	68.111/RGB-U//WARD RESEL/3/STIL/4/AE.SQUARROSA (700)	1.4	R	
39	68.111/RGB-U//WARD RESEL/3/STIL/4/AE.SQUARROSA (768)	1.1	R	
40	68.111/RGB-U//WARD RESEL/3/STIL/4/AE.SQUARROSA (1010)	1.9	MR	
41	**68.111/RGB-U//WARD/3/FGO/4/RABI***	3.2	MS	31
42	68.111/RGB-U//WARD/3/FGO/4/RABI/5/AE.SQUARROSA (191)	1.2	R	
43	68.111/RGB-U//WARD/3/FGO/4/RABI/5/AE.SQUARROSA (878)	1.3	R	
44	68.111/RGB-U//WARD/3/FGO/4/RABI/5/AE.SQUARROSA (878)	1.1	R	
45	68.111/RGB-U//WARD/3/FGO/4/RABI/5/AE.SQUARROSA (882)	1.1	R	
46	68.111/RGB-U//WARD/3/FGO/4/RABI/5/AE.SQUARROSA (905)	1.3	R	
47	68.111/RGB-U//WARD/3/FGO/4/RABI/5/AE.SQUARROSA (809)	1.3	R	
48	68.111/RGB-U//WARD/3/FGO/4/RABI/5/AE.SQUARROSA (809)	1.1	R	
49	68.111/RGB-U//WARD/3/FGO/4/RABI/5/AE.SQUARROSA (809)	1.3	R	
50	68.111/RGB-U//WARD/3/FGO/4/RABI/5/AE.SQUARROSA (878)	1.6	MR	
51	68.111/RGB-U//WARD/3/FGO/4/RABI/5/AE.SQUARROSA (878)	1.8	MR	
52	68.111/RGB-U//WARD/3/FGO/4/RABI/5/AE.SQUARROSA (878)	1.5	R	
53	68.111/RGB-U//WARD/3/FGO/4/RABI/5/AE.SQUARROSA (878)	2.2	MR	
54	68.111/RGB-U//WARD/3/FGO/4/RABI/5/AE.SQUARROSA (878)	1.2	R	
55	68.111/RGB-U//WARD/3/FGO/4/RABI/5/AE.SQUARROSA (878)	3.3	MS	
56	68.111/RGB-U//WARD/3/FGO/4/RABI/5/AE.SQUARROSA (878)	2.5	MR	
57	68.111/RGB-U//WARD/3/FGO/4/RABI/5/AE.SQUARROSA (1050)	1.2	R	
58	68.111/RGB-U//WARD/3/FGO/4/RABI/5/AE.SQUARROSA (719)	2.4	MR	
59	68.111/RGB-U//WARD/3/FGO/4/RABI/5/AE.SQUARROSA (720)	2.0	MR	
60	68.111/RGB-U//WARD/3/FGO/4/RABI/5/AE.SQUARROSA (768)	1.0	R	
61	68.111/RGB-U//WARD/3/FGO/4/RABI/5/AE.SQUARROSA (778)	1.4	R	
62	68.111/RGB-U//WARD/3/FGO/4/RABI/5/AE.SQUARROSA (788)	1.8	MR	

63	68.111/RGB-U//WARD/3/FGO/4/RABI/5/AE.SQUARROSA (974)	1.7	MR	
64	68.111/RGB-U//WARD/3/FGO/4/RABI/5/AE.SQUARROSA (661)	1.1	R	
65	68.111/RGB-U//WARD/3/FGO/4/RABI/5/AE.SQUARROSA (701)	1.0	R	
66	68.111/RGB-U//WARD/3/FGO/4/RABI/5/AE.SQUARROSA (709)	1.4	R	
67	68.111/RGB-U//WARD/3/FGO/4/RABI/5/AE.SQUARROSA (710)	.	.	
68	68.111/RGB-U//WARD/3/FGO/4/RABI/5/AE.SQUARROSA (784)	1.2	R	
69	68.111/RGB-U//WARD/3/FGO/4/RABI/5/AE.SQUARROSA (504)	1.2	R	
70	68.111/RGB-U//WARD/3/FGO/4/RABI/5/AE.SQUARROSA (675)	1.2	R	
71	68.111/RGB-U//WARD/3/FGO/4/RABI/5/AE.SQUARROSA (1010)	1.3	R	
72	68.111/RGB-U//WARD/3/FGO/4/RABI/5/AE.SQUARROSA (1093)	1.3	R	
73	**68112/WARD***	2.3	MR	4
74	68112/WARD//AE.SQUARROSA (369)	1.3	R	
75	68112/WARD//AE.SQUARROSA (369)	1.6	MR	
76	68112/WARD//AE.SQUARROSA (369)	1.2	R	
77	68112/WARD//AE.SQUARROSA (369)	2.0	MR	
78	**6973/WARD.7463//74110***	2.3	MR	3
79	6973/WARD.7463//74110/3/AE.SQUARROSA (35A)	2.6	MS	
80	6973/WARD.7463//74110/3/AE.SQUARROSA (665)	1.3	R	
81	6973/WARD.7463//74110/3/AE.SQUARROSA (438)	1.2	R	
82	**ACONCHI 89***	1.7	MR	4
83	ACO89/AE.SQUARROSA (178)	1.5	R	
84	ACO89/AE.SQUARROSA (282)	1.9	MR	
85	ACO89/AE.SQUARROSA (290)	2.5	MR	
86	ACO89/AE.SQUARROSA (309)	2.2	MR	
87	**ALG86/4/FGO/PALES//MEXI_1/3/RUFF/FGO/5/ENTE***	1.8	MR	3
88	ALG86/4/FGO/PALES//MEXI_1/3/RUFF/FGO/5/ENTE/6/AE.SQUARROSA (389)	2.4	MR	
89	ALG86/4/FGO/PALES//MEXI_1/3/RUFF/FGO/5/ENTE/6/AE.SQUARROSA (451)	3.4	MS	
90	ALG86/4/FGO/PALES//MEXI_1/3/RUFF/FGO/5/ENTE/6/AE.SQUARROSA (723)	2.0	MR	
91	**ALTAR 84***	1.3	R	20
92	ALTAR 84/AE.SQUARROSA (188)	1.2	R	
93	ALTAR 84/AE.SQUARROSA (191)	1.1	R	
94	ALTAR 84/AE.SQUARROSA (198)	1.0	R	
95	ALTAR 84/AE.SQUARROSA (220)	2.3	MR	
96	ALTAR 84/AE.SQUARROSA (221)	1.0	R	

97	ALTAR 84/AE.SQUARROSA (223)	1.3	R	
98	ALTAR 84/AE.SQUARROSA (224)	1.0	R	
99	ALTAR 84/AE.SQUARROSA (224)	1.9	MR	
100	ALTAR 84/AE.SQUARROSA (224)	2.2	MR	
101	ALTAR 84/AE.SQUARROSA (291)	1.9	MR	
102	ALTAR 84/AE.SQUARROSA(Y86-87 S401)	1.2	R	
103	ALTAR 84/AE.SQUARROSA (333)	3.9	S	
104	ALTAR 84/AE.SQUARROSA (507)	1.6	MR	
105	ALTAR 84/AE.SQUARROSA (174)	1.5	R	
106	ALTAR 84/AE.SQUARROSA (1012)	1.8	MR	
107	ALTAR 84/AE.SQUARROSA (244)	1.9	MR	
108	ALTAR 84/AE.SQUARROSA (319)	1.3	R	
109	ALTAR 84/AE.SQUARROSA (531)	2.5	MR	
110	ALTAR 84/AE.SQUARROSA (539)	4.1	S	
111	ALTAR 84/AE.SQUARROSA (793)	1.3	R	
112	**ARLIN_1***	2.4	MR	13
113	ARLIN/AE.SQUARROSA (283)	1.6	R	
114	ARLIN/AE.SQUARROSA (317)	2.1	MR	
115	ARLIN_1/AE.SQUARROSA (333)	1.8	MR	
116	ARLIN/AE.SQUARROSA (410)	2.0	MR	
117	ARLIN_1/AE.SQUARROSA (536)	1.5	R	
118	ARLIN_1/AE.SQUARROSA (1018)	2.6	MS	
119	AE.SQUARROSA (1031)/ARLIN_1	1.1	R	
120	ARLIN_1/AE.SQUARROSA (310)	1.1	R	
121	ARLIN_1/AE.SQUARROSA (320)	1.4	R	
122	ARLIN_1/AE.SQUARROSA (368)	1.3	R	
123	ARLIN_1/AE.SQUARROSA (430)	1.3	R	
124	ARLIN_1/AE.SQUARROSA (335)	1.2	R	
125	ARLIN_1/AE.SQUARROSA (802)	1.3	R	
126	**BOTNO***	3.4	MS	1
127	BOTNO/AE.SQUARROSA (617)	1.5	R	
128	**CERCETA***	1.3	R	54
129	CETA/AE.SQUARROSA (230)	1.3	R	
130	CETA/AE.SQUARROSA (783)	1.1	R	

131	CETA/AE.SQUARROSA (895)	1.2	R
132	CETA/AE.SQUARROSA (895)	1.5	R
133	CETA/AE.SQUARROSA (796)	1.9	MR
134	CETA/AE.SQUARROSA (174)	1.3	R
135	CETA/AE.SQUARROSA (499)	1.3	R
136	CETA/AE.SQUARROSA (525)	1.1	R
137	CETA/AE.SQUARROSA (540)	1.9	MR
138	CETA/AE.SQUARROSA (1016)	1.9	MR
139	CETA/AE.SQUARROSA (1027)	1.5	R
140	CETA/AE.SQUARROSA (1030)	2.0	MR
141	CETA/AE.SQUARROSA (166)	1.9	MR
142	CETA/AE.SQUARROSA (187)	1.6	MR
143	CETA/AE.SQUARROSA (244)	3.1	MS
144	CETA/AE.SQUARROSA (262)	1.2	R
145	CETA/AE.SQUAROOSA (263)	2.1	MR
146	CETA/AE.SQUARROSA (371)	1.4	R
147	CETA/AE.SQUARROSA (391)	2.8	MS
148	CETA/AE.SQUARROSA (445)	1.5	R
149	CETA/AE.SQUARROSA (450)	2.4	MR
150	CETA/AE.SQUARROSA (485)	2.7	MS
151	CETA/AE.SQUARROSA (530)	2.6	MS
152	CETA/AE.SQUARROSA (533)	2.5	MR
153	CETA/T.URARTU (557)	1.3	R
154	CETA/AE.SQUARROSA (1018)	2.0	MR
155	CETA/AE.SQUARROSA (1026)	1.4	R
156	CETA/AE.SQUARROSA (1031)	1.4	R
157	CETA/AE.SQUARROSA (1036)	1.8	MR
158	CETA/AE.SQUARROSA (1038)	1.9	MR
159	CETA/AE.SQUARROSA (1043)	1.6	MR
160	CETA/AE.SQUARROSA (1053)	1.4	R
161	CETA/AE.SQUARROSA (246)	2.1	MR
162	CETA/AE.SQUARROSA (496)	1.5	R
163	CETA/AE.SQUARROSA (506)	3.3	MS
164	CETA/AE.SQUARROSA (539)	3.6	S

165	CETA/AE.SQUARROSA (541)	3.3	MS	
166	CETA/AE.SQUARROSA (231)	1.3	R	
167	CETA/AE.SQUARROSA (335)	1.3	R	
168	CETA/AE.SQUARROSA (356)	2.0	MR	
169	CETA/AE.SQUARROSA (1047)	2.1	MR	
170	CETA/AE.SQUARROSA (615)	1.4	R	
171	CETA/AE.SQUARROSA (629)	1.3	R	
172	CETA/AE.SQUARROSA (750)	1.2	R	
173	CETA/AE.SQUARROSA (1090)	1.7	MR	
174	CETA/AE.SQUARROSA (248)	1.4	R	
175	CETA/AE.SQUARROSA (310)	1.3	R	
176	CETA/AE.SQUARROSA (418)	1.2	R	
177	CETA/AE.SQUARROSA (442)	2.0	MR	
178	CETA/AE.SQUARROSA (681)	1.4	R	
179	CETA/AE.SQUARROSA (682)	1.0	R	
180	CETA/AE.SQUARROSA (683)	1.4	R	
181	CETA/AE.SQUARROSA (684)	1.5	R	
182	CETA/AE.SQUARROSA (1073)	1.6	MR	
183	**CHEN_7***	2.6	MS	1
184	CHEN_7/AE.SQUARROSA (429)	2.3	MR	
185	**CPI8/GEDIZ/3/GOO//ALB/CRA***	2.3	MR	31
186	CPI8/GEDIZ/3/GOO//ALB/CRA/4/AE.SQUARROSA (193)	1.4	R	
187	CPI8/GEDIZ/3/GOO//ALB/CRA/4/AE.SQUARROSA (196)	1.3	R	
188	CPI8/GEDIZ/3/GOO//ALB/CRA/4/AE.SQUARROSA (205)	1.6	MR	
189	CPI8/GEDIZ/3/GOO//ALB/CRA/4/AE.SQUARROSA (208)	1.3	R	
190	CPI8/GEDIZ/3/GOO//ALB/CRA/4/AE.SQUARROSA (215)	1.3	R	
191	CPI8/GEDIZ/3/GOO//ALB/CRA/4/AE.SQUARROSA (629)	1.5	R	
192	CPI8/GEDIZ/3/GOO//ALB/CRA/4/AE.SQUARROSA (633)	2.3	MR	
193	CPI8/GEDIZ/3/GOO//ALB/CRA/4/AE.SQUARROSA (637)	1.4	R	
194	CPI8/GEDIZ/3/GOO//ALB/CRA/4/AE.SQUARROSA (358)	1.8	MR	
195	CPI8/GEDIZ/3/GOO//ALB/CRA/4/AE.SQUARROSA (334)	1.3	R	
196	CPI8/GEDIZ/3/GOO//ALB/CRA/4/AE.SQUARROSA (409)	1.6	MR	
197	CPI8/GEDIZ/3/GOO//ALB/CRA/4/AE.SQUARROSA (184)	1.8	MR	
198	CPI8/GEDIZ/3/GOO//ALB/CRA/4/AE.SQUARROSA (244)	2.5	MR	

199	CPI8/GEDIZ/3/GOO//ALB/CRA/4/AE.SQUARROSA (273)	2.6	MS	
200	CPI8/GEDIZ/3/GOO//ALB/CRA/4/AE.SQUARROSA (296)	3.0	MS	
201	CPI8/GEDIZ/3/GOO//ALB/CRA/4/AE.SQUARROSA (305)	2.9	MS	
202	CPI8/GEDIZ/3/GOO//ALB/CRA/4/AE.SQUARROSA (439)	1.9	MR	
203	CPI8/GEDIZ/3/GOO//ALB/CRA/4/AE.SQUARROSA (461)	2.2	MR	
204	CPI8/GEDIZ/3/GOO//ALB/CRA/4/AE.SQUARROSA (533)	2.2	MR	
205	CPI8/GEDIZ/3/GOO//ALB/CRA/4/AE.SQUARROSA (1018)	1.4	R	
206	CPI8/GEDIZ/3/GOO//ALB/CRA/4/AE.SQUARROSA (1021)	1.5	R	
207	CPI8/GEDIZ/3/GOO//ALB/CRA/4/AE.SQUARROSA (1026)	1.3	R	
208	CPI8/GEDIZ/3/GOO//ALB/CRA/4/AE.SQUARROSA (1029)	1.6	MR	
209	CPI8/GEDIZ/3/GOO//ALB/CRA/4/AE.SQUARROSA (1031)	2.5	MR	
210	AE.SQUARROSA (1043)/4/CPI8/GEDIZ/3/GOO//ALB/CRA	1.3	R	
211	CPI8/GEDIZ/3/GOO//ALB/CRA/4/AE.SQUARROSA (227)	1.4	R	
212	CPI8/GEDIZ/3/GOO//ALB/CRA/4/AE.SQUARROSA (1017)	2.1	MR	
213	CPI8/GEDIZ/3/GOO//ALB/CRA/4/AE.SQUARROSA (188)	1.5	R	
214	CPI8/GEDIZ/3/GOO//ALB/CRA/4/AE.SQUARROSA (659)	1.7	MR	
215	CPI8/GEDIZ/3/GOO//ALB/CRA/4/AE.SQUARROSA (684)	1.3	R	
216	CPI8/GEDIZ/3/GOO//ALB/CRA/4/AE.SQUARROSA (698)	2.3	MR	
217	**CROC_1***	1.4	R	30
218	CROC_1/AE.SQUARROSA (168)	1.0	R	
219	CROC_1/AE.SQUARROSA (205)	1.3	R	
220	CROC_1/AE.SQUARROSA (210)	1.9	MR	
221	CROC_1/AE.SQUARROSA (210)	1.8	MR	
222	CROC_1/AE.SQUARROSA (210)	1.4	R	
223	CROC_1/AE.SQUARROSA (213)	1.3	R	
224	CROC_1/AE.SQUARROSA (215)	1.0	R	
225	CROC_1/AE.SQUARROSA (224)	1.0	R	
226	CROC_1/AE.SQUARROSA (224)	1.0	R	
227	CROC_1/AE.SQUARROSA (224)	1.1	R	
228	CROC_1/AE.SQUARROSA (224)	1.0	R	
229	CROC_1/AE.SQUARROSA (662)	1.3	R	
230	CROC_1/AE.SQUARROSA (725)	1.2	R	
231	CROC_1/AE.SQUARROSA (826)	1.7	MR	
232	CROC_1/AE.SQUARROSA (886)	1.3	R	

233	CROC_1/AE.SQUARROSA (518)	1.3	R	
234	CROC_1/AE.SQUARROSA (298)	2.4	MR	
235	CROC_1/AE.SQUARROSA (333)	1.6	MR	
236	CROC_1/AE.SQUARROSA (170)	1.6	MR	
237	CROC_1/AE.SQUARROSA (177)	1.4	R	
238	CROC_1/AE.SQUARROSA (256)	2.1	MR	
239	CROC_1/AE.SQUARROSA (275)	1.7	MR	
240	CROC_1/AE.SQUARROSA (516)	1.1	R	
241	CROC_1/AE.SQUARROSA (517)	1.3	R	
242	CROC_1/AE.SQUARROSA (493)	1.9	MR	
243	CROC_1/AE.SQUARROSA (176)	2.0	MR	
244	CROC_1/AE.SQUARROSA (229)	1.9	MR	
245	CROC_1/AE.SQUARROSA (310)	1.5	R	
246	CROC_1/AE.SQUARROSA (239)	2.0	MR	
247	CROC_1/AE.SQUARROSA (397)	1.9	MR	
248	**D67.2/PARANA 66.270***	3.1	MS	13
249	D67.2/PARANA 66.270//AE.SQUARROSA (211)	1.5	R	
250	D67.2/PARANA 66.270//AE.SQUARROSA (213)	1.5	R	
251	D67.2/PARANA 66.270//AE.SQUARROSA (218)	1.4	R	
252	D67.2/PARANA 66.270//AE.SQUARROSA (220)	1.4	R	
253	D67.2/PARANA 66.270//AE.SQUARROSA (221)	1.5	R	
254	D67.2/PARANA 66.270//AE.SQUARROSA (222)	1.1	R	
255	D67.2/PARANA 66.270//AE.SQUARROSA (223)	1.2	R	
256	D67.2/PARANA 66.270//AE.SQUARROSA (633)	1.1	R	
257	D67.2/PARANA 66.270//AE.SQUARROSA (246)	1.3	R	
258	D67.2/PARANA 66.270//AE.SQUARROSA (657)	1.3	R	
259	D67.2/PARANA 66.270//AE.SQUARROSA (634)	1.6	MR	
260	D67.2/PARANA 66.270//AE.SQUARROSA (668)	1.0	R	
261	D67.2/PARANA 66.270//AE.SQUARROSA (1148)	1.3	R	
262	**DECOY 1***	2.5	MR	30
263	DOY1/AE.SQUARROSA (188)	1.1	R	
264	DOY1/AE.SQUARROSA (216)	2.1	MR	
265	DOY1/AE.SQUARROSA (446)	1.8	MR	
266	DOY1/AE.SQUARROSA (447)	2.8	MS	

267	DOY1/AE.SQUARROSA (488)	1.7	MR	
268	DOY1/AE.SQUARROSA (510)	1.5	R	
269	DOY1/AE.SQUARROSA (515)	1.8	MR	
270	DOY1/AE.SQUARROSA (415)	2.7	MS	
271	DOY1/AE.SQUARROSA (428)	1.5	R	
272	DOY1/AE.SQUARROSA (507)	1.2	R	
273	DOY1/AE.SQUARROSA (532)	2.1	MR	
274	DOY1/AE.SQUARROSA (177)	2.1	MR	
275	DOY1/AE.SQUARROSA (255)	1.7	MR	
276	DOY1/AE.SQUARROSA (258)	1.3	R	
277	DOY1/AE.SQUARROSA (267)	1.6	MR	
278	DOY1/AE.SQUARROSA (322)	1.1	R	
279	DOY1/AE.SQUARROSA (334)	1.4	R	
280	DOY1/AE.SQUARROSA (516)	2.0	MR	
281	DOY1/AE.SQUARROSA (517)	1.5	R	
282	DOY1/AE.SQUARROSA (1016)	1.3	R	
283	DOY1/AE.SQUARROSA (1024)	1.7	MR	
284	DOY1/AE.SQUARROSA (1018)	2.6	MS	
285	DOY1/AE.SQUARROSA (1026)	1.3	R	
286	DOY1/AE.SQUARROSA (1029)	1.3	R	
287	AE.SQUARROSA (1043)/DOY1	2.0	MR	
288	AE.SQUARROSA (1026)/DOY1	2.1	MR	
289	DOY1/AE.SQUARROSA (295)	2.4	MR	
290	DOY1/AE.SQUARROSA (360)	2.5	MR	
291	DOY1/AE.SQUARROSA (540)	3.5	MS	
292	DOY1/AE.SQUARROSA (632)	1.3	R	
293	**DVERD_2***	1.5	R	13
294	DVERD_2/AE.SQUARROSA (214)	1.3	R	
295	DVERD_2/AE.SQUARROSA (221)	1.3	R	
296	DVERD_2/AE.SQUARROSA (247)	1.1	R	
297	DVERD_2/AE.SQUARROSA (247)	1.5	R	
298	DVERD_2/AE.SQUARROSA (333)	1.8	MR	
299	DVERD_2/AE.SQUARROSA (507)	2.3	MR	
300	DVERD_2/AE.SQUARROSA (1022)	1.3	R	

301	DVERD_2/T.URARTU (545)	1.8	MR	
302	DVERD_2/AE.SQUARROSA (1026)	1.8	MR	
303	DVERD_2/AE.SQUARROSA (1029)	1.7	MR	
304	DVERD_2/AE.SQUARROSA (1031)	1.8	MR	
305	AE.SQUARROSA (1029)/DVERD_2	1.9	MR	
306	AE.SQUARROSA (1031)/DVERD_2	2.1	MR	
307	**FALCIN_1***	1.0	R	5
308	FALCIN/AE.SQUARROSA (312)	1.6	MR	
309	FALCIN/AE.SQUARROSA (389)	2.2	MR	
310	FALCIN_1/AE.SQUARROSA (176)	2.4	MR	
311	FALCIN_1/AE.SQUARROSA (197)	1.8	MR	
312	FALCIN_1/AE.SQUARROSA (1073)	1.3	R	
313	**FGO/USA2111***	1.3	R	1
314	FGO/USA2111//AE.SQUARROSA (658)	1.0	R	
315	**GAN***	2.0	MR	39
316	GAN/AE.SQUARROSA (201)	1.5	R	
317	GAN/AE.SQUARROSA (446)	3.3	MS	
318	GAN/AE.SQUARROSA (522)	2.1	MR	
319	GAN/AE.SQUARROSA (180)	1.0	R	
320	GAN/AE.SQUARROSA (257)	1.1	R	
321	GAN/AE.SQUARROSA (408)	1.0	R	
322	GAN/AE.SQUARROSA (890)	1.0	R	
323	GAN/AE.SQUARROSA (163)	1.8	MR	
324	GAN/AE.SQUARROSA (182)	1.6	MR	
325	GAN/AE.SQUARROSA (264)	1.4	R	
326	GAN/AE.SQUARROSA (267)	1.5	R	
327	GAN/AE.SQUARROSA (268)	1.3	R	
328	GAN/AE.SQUARROSA (285)	1.0	R	
329	GAN/AE.SQUARROSA (413)	1.1	R	
330	GAN/AE.SQUARROSA (459)	1.3	R	
331	GAN/AE.SQUARROSA (206)	1.4	R	
332	GAN/AE.SQUARROSA (231)	1.3	R	
333	GAN/AE.SQUARROSA (233)	1.6	MR	
334	GAN/AE.SQUARROSA (296)	1.5	R	

335	GAN/AE.SQUARROSA (300)	1.6	MR	
336	GAN/AE.SQUARROSA (335)	1.1	R	
337	GAN/AE.SQUARROSA (536)	1.7	MR	
338	GAN/AE.SQUARROSA (620)	2.4	MR	
339	GAN/AE.SQUARROSA (621)	2.7	MS	
340	GAN/AE.SQUARROSA (623)	1.0	R	
341	GAN/AE.SQUARROSA (624)	1.0	R	
342	GAN/AE.SQUARROSA (633)	1.2	R	
343	GAN/AE.SQUARROSA (638)	1.3	R	
344	GAN/AE.SQUARROSA (658)	1.1	R	
345	GAN/AE.SQUARROSA (668)	1.6	MR	
346	GAN/AE.SQUARROSA (643)	2.7	MS	
347	GAN/AE.SQUARROSA (741)	1.0	R	
348	GAN/AE.SQUARROSA (479)	1.0	R	
349	GAN/AE.SQUARROSA (680)	1.3	R	
350	GAN/AE.SQUARROSA (721)	1.2	R	
351	GAN/AE.SQUARROSA (735)	1.1	R	
352	GAN/AE.SQUARROSA (768)	1.4	R	
353	GAN/AE.SQUARROSA (779)	1.5	R	
354	GAN/AE.SQUARROSA (1080)	1.5	R	
355	**GARZA/BOY***	1.3	R	7
356	GARZA/BOY//AE.SQUARROSA (271)	1.5	R	
357	GARZA/BOY//AE.SQUARROSA (286)	1.3	R	
358	GARZA/BOY//AE.SQUARROSA (307)	2.9	MS	
359	GARZA/BOY//AE.SQUARROSA (311)	2.2	MR	
360	GARZA/BOY//AE.SQUARROSA (350)	1.7	MR	
361	GARZA/BOY//AE.SQUARROSA (439)	1.8	MR	
362	GARZA/BOY//AE.SQUARROSA (764)	2.0	MR	
363	**GREEN***	1.1	R	1
364	GREEN/AE.SQUARROSA (458)	1.4	R	
365	**KAPUDE_1***	2.2	MR	1
366	KAPUDE/AE.SQUARROSA (175)	1.8	MR	
367	**LARU***	1.3	R	4
368	LARU/AE.SQUARROSA (309)	1.5	R	

369	LARU/AE.SQUARROSA (309)	1.4	R	
370	LARU/AE.SQUARROSA (TA2459)	1.4	R	
371	LARU/AE.SQUARROSA (333)	1.6	MR	
372	**LCK59.61***	3.0	MS	2
373	LCK59.61/AE.SQUARROSA (308)	1.4	R	
374	LCK59.61/AE.SQUARROSA (783)	2.1	MR	
375	**LOCAL RED***	1.9	MR	7
376	LOCAL RED/AE.SQUARROSA (219)	2.4	MR	
377	LOCAL RED/AE.SQUARROSA (220)	2.3	MR	
378	LOCAL RED/AE.SQUARROSA (221)	2.0	MR	
379	LOCAL RED/AE.SQUARROSA (222)	2.1	MR	
380	LOCAL RED/AE.SQUARROSA (223)	2.3	MR	
381	LOCAL RED/AE.SQUARROSA (449)	1.3	R	
382	LOCAL RED/AE.SQUARROSA (189)	2.8	MS	
383	**RABI//GS/CRA***	1.1	R	4
384	RABI//GS/CRA/3/AE.SQUARROSA (190)	1.0	R	
385	RABI//GS/CRA/3/AE.SQUARROSA (891)	1.3	R	
386	RABI//GS/CRA/3/AE.SQUARROSA (904)	1.8	MR	
387	RABI//GS/CRA/3/AE.SQUARROSA (457)	1.2	R	
388	**RASCON***	1.1	R	2
389	RASCON/AE.SQUARROSA (312)	1.5	R	
390	RASCON/AE.SQUARROSA (367)	2.3	MR	
391	**ROK/KML***	1.0	R	4
392	ROK/KML//AE.SQUARROSA (214)	1.3	R	
393	ROK/KML//AE.SQUARROSA (295)	2.5	MR	
394	ROK/KML//AE.SQUARROSA (333)	1.5	R	
395	ROK/KML//AE.SQUARROSA (507)	2.3	MR	
396	**SCAUP***	2.0	MR	3
397	SCA/AE.SQUARROSA (493)	2.6	MS	
398	SCA/AE.SQUARROSA (248)	2.1	MR	
399	SCA/AE.SQUARROSA (409)	1.0	R	
400	**SCOOP_1***	1.2	R	3
401	SCOOP_1/AE.SQUARROSA (358)	1.3	R	
402	SCOOP_1/AE.SQUARROSA (407)	1.1	R	

403	SCOOP_1/AE.SQUARROSA (659)	1.1	R	
404	**SCOT/MEXI_1***	1.0	R	1
405	SCOT/MEXI_1//AE.SQUARROSA (186)	1.3	R	
406	**SHAG_22***	1.3	R	6
407	SHAG_22/AE.SQUARROSA (227)	1.3	R	
408	SHAG_22/AE.SQUARROSA (319)	1.6	MR	
409	SHAG_22/AE.SQUARROSA (530)	1.8	MR	
410	SHAG_22/AE.SQUARROSA (537)	2.8	MS	
411	SHAG_22/AE.SQUARROSA (539)	2.5	MR	
412	SHAG_22/AE.SQUARROSA (1101)	1.2	R	
413	**SNIPE/YAV79//DACK/TEAL***	1.0	R	7
414	SNIPE/YAV79//DACK/TEAL/3/AE.SQUARROSA (411)	1.6	MR	
415	SNIPE/YAV79//DACK/TEAL/3/AE.SQUARROSA (904)	1.0	R	
416	SNIPE/YAV79//DACK/TEAL/3/AE.SQUARROSA (528)	1.3	R	
417	SNIPE/YAV79//DACK/TEAL/3/AE.SQUARROSA (628)	1.4	R	
418	SNIPE/YAV79//DACK/TEAL/3/AE.SQUARROSA (629)	1.3	R	
419	SNIPE/YAV79//DACK/TEAL/3/AE.SQUARROSA (633)	1.3	R	
420	SNIPE/YAV79//DACK/TEAL/3/AE.SQUARROSA (700)	1.6	MR	
421	**SORA***	1.1	R	14
422	SORA/AE.SQUARROSA (191)	1.4	R	
423	SORA/AE.SQUARROSA (192)	1.4	R	
424	SORA/AE.SQUARROSA (192)	1.2	R	
425	SORA/AE.SQUARROSA (207)	1.5	R	
426	SORA/AE.SQUARROSA (208)	1.4	R	
427	SORA/AE.SQUARROSA (211)	1.4	R	
428	SORA/AE.SQUARROSA (215)	1.6	MR	
429	SORA/AE.SQUARROSA (323)	1.4	R	
430	SORA/AE.SQUARROSA (939)	2.9	MS	
431	SORA/AE.SQUARROSA (617)	1.4	R	
432	SORA/AE.SQUARROSA (625)	2.0	MR	
433	SORA/AE.SQUARROSA (442)	1.5	R	
434	SORA/AE.SQUARROSA (469)	1.1	R	
435	SORA/AE.SQUARROSA (684)	2.2	MR	
436	**STY,DR/CELTA//PALS/3/SRN_5***	1.2	R	2

437	STY,DR/CELTA//PALS/3/SRN_5/4/AE.SQUARROSA (277)	1.3	R	
438	STY,DR/CELTA//PALS/3/SRN_5/4/AE.SQUARROSA (502)	1.1	R	
439	**TK SN1081***	3.2	MS	3
440	TK SN1081/AE.SQUARROSA (222)	1.2	R	
441	TK SN1081/AE.SQUARROSA (222)	1.0	R	
442	TK SN1081/AE.SQUARROSA (690)	1.7	MR	
443	**YAR***	2.5	MR	4
444	YAR/AE.SQUARROSA (493)	2.7	MS	
445	YAR/AE.SQUARROSA (783)	1.5	R	
446	YAR/AE.SQUARROSA (809)	1.0	R	
447	YAR/AE.SQUARROSA (518)	1.1	R	
448	**YARMUK***	2.1	MR	4
449	YUK/AE.SQUARROSA (217)	2.1	MR	
450	YUK/AE.SQUARROSA (434)	1.4	R	
451	YUK/AE.SQUARROSA (784)	1.6	MR	
452	YUK/AE.SQUARROSA (864)	1.2	R	
453	**YAV_2/TEZ***	2.3	MR	12
454	YAV_2/TEZ//AE.SQUARROSA (249)	2.0	MR	
455	YAV_2/TEZ//AE.SQUARROSA (249)	1.4	R	
456	YAV_2/TEZ//AE.SQUARROSA (249)	1.3	R	
457	YAV_2/TEZ//AE.SQUARROSA (249)	1.2	R	
458	YAV_2/TEZ//AE.SQUARROSA (249)	1.3	R	
459	YAV_2/TEZ//AE.SQUARROSA (249)	1.7	MR	
460	YAV_2/TEZ//AE.SQUARROSA (435)	1.5	R	
461	YAV_2/TEZ//AE.SQUARROSA (437)	1.5	R	
462	YAV_2/TEZ//AE.SQUARROSA (882)	1.4	R	
463	YAV_2/TEZ//AE.SQUARROSA (746)	1.1	R	
464	YAV_2/TEZ//AE.SQUARROSA (721)	1.3	R	
465	YAV_2/TEZ//AE.SQUARROSA (1093)	1.3	R	
466	Lines without durum wheat parents in this study			
467	YAV79//DACK/RABI/3/SNIPE/4/AE.SQUARROSA (460)	1.5	R	
468	YAV79//DACK/RABI/3/SNIPE/4/AE.SQUARROSA (460)	1.1	R	
469	YAV79//DACK/RABI/3/SNIPE/4/AE.SQUARROSA (477)	2.3	MR	
470	YAV79//DACK/RABI/3/SNIPE/4/AE.SQUARROSA (477)	1.5	R	

471	SRN/AE.SQUARROSA (358)	1.2	R
472	YAV79//DACK/RABI/3/SNIPE/4/AE.SQUARROSA (381)	1.1	R
473	YAV79//DACK/RABI/3/SNIPE/4/AE.SQUARROSA (397)	1.2	R
474	YAV79//DACK/RABI/3/SNIPE/4/AE.SQUARROSA (443)	1.3	R
475	YAV79//DACK/RABI/3/SNIPE/4/AE.SQUARROSA (490)	1.3	R
476	BACANORA T 88	2.6	MS
477	CADO/BOOMER_33//AE.SQUARROSA (651)	.	.
478	CADO/BOOMER_33//AE.SQUARROSA (949)	1.6	MR
479	CADO/BOOMER_33//AE.SQUARROSA (504)	2.1	MR
480	DUKEM_12/2*RASCON_21//AE.SQUARROSA (1100)	1.1	R
481	KUCUK/AE.SQUARROSA (458)	1.3	R
482	KUCUK/AE.SQUARROSA (1080)	1.9	MR
483	KUCUK/AE.SQUARROSA (640)	1.3	R
484	DUKEM_12/2*RASCON_21//AE.SQUARROSA (1090)	1.5	R
	Check resistent Chirya 3	1.4	R
	Check susceptible Sonalika	4.0	S
	Check susceptible Ciano T79	4.0	S
	Check moderately susceptible Francolin	2.8	MS

* Durum wheat parents.

**Averaged spot blotch reaction of each genotype of SHW (six replications) and durum wheat parents (eight replications)
