# Supplementary material for: Genome-Wide Association Study for Spot Blotch Resistance in Synthetic Hexaploid Wheat
Source: Genes (Basel). 2022 Aug 4;13(8):1387. doi: 10.3390/genes13081387 (PMC9407756; doi:10.3390/genes13081387)
Supplement: Supplementary file 1 [file genes-13-01387-s001.zip › Supplementary Table S2.pdf]

Table S2. Significant markers for seedling resistance to spot blotch when aligned to the physical map of Chinese spring (IWGSC RefSeqV.1.0). Chromosome (Chr.), marker ID, allele ID, physical position, F statistics, Probability (Prob), Marker R^2, $-\log_{10}$ (p-value) and the effect of allele substitution are given for each marker.

Chr.	Marker ID	Allele ID	Position	F statistic	Prob	Marker R^2	$-\log_{10}$ p-value	Effect of allele substitution (genotype effect)
1B	1145134	1145134|F|0-37:T>C-37:T>C	406039536	11.33	1.64×10^{-5}	0.06	4.79	-0.05
1D	1125496	1125496|F|0-23:T>C-23:T>C	416590812	13.08	3.36×10^{-4}	0.03	3.47	NaN
2A	1144884	1144884|F|0-29:C>T-29:C>T	583026867	13.33	2.50×10^{-6}	0.07	5.60	0.02
2D	1089634	1089634|F|0-38:A>C-38:A>C	509231294	10.66	3.10×10^{-5}	0.05	4.51	0.03
2D	2243785	2243785|F|0-27:T>C-27:T>C	32640660	8.49	2.46×10^{-4}	0.04	3.61	-0.18
2D	1122278	1122278|F|0-8:C>A-8:C>A	21621448	7.21	8.39×10^{-4}	0.04	3.08	-0.14
3A	2279238	2279238|F|0-47:C>T-47:C>T	474554774	10.10	5.27×10^{-5}	0.05	4.28	0.33
3A	1019955	1019955|F|0-55:A>G-55:A>G	474447292	7.11	9.28×10^{-4}	0.03	3.03	-0.46
3B	1283998	1283998|F|0-27:G>A-27:G>A	593544135	10.68	3.04×10^{-5}	0.05	4.52	-0.02
3D	1011260	1011260|F|0-43:A>T-43:A>T	520678096	8.80	1.83×10^{-4}	0.04	3.74	-0.05
4A	1351280	1351280|F|0-50:G>T-50:G>T	629433955	8.83	1.78×10^{-4}	0.04	3.75	-0.06
5A	3570010	3570010|F|0-29:G>A-29:G>A	521764788	13.73	2.40×10^{-4}	0.03	3.62	NaN
5A	1046932	1046932|F|0-42:G>A-42:G>A	622389460	12.13	5.52×10^{-4}	0.03	3.26	NaN
5D	1086529	1086529|F|0-68:G>T-68:G>T	410253879	8.41	2.65×10^{-4}	0.04	3.58	0.21
5D	100016153	100016153|F|0-26:G>A-26:G>A	232599413	7.10	9.35×10^{-4}	0.04	3.03	0.32
6D	1698662	1698662|F|0-37:G>C-37:G>C	42940457	9.30	1.13×10^{-4}	0.05	3.95	-0.27
7A	990293	990293|F|0-7:G>A-7:G>A	621213334	10.66	3.11×10^{-5}	0.05	4.51	-0.03
7A	4002611	4002611|F|0-59:C>G-59:C>G	7938756	9.55	8.88×10^{-5}	0.05	4.05	-0.04
7D	22765212	22765212|F|0-33:C>A-33:C>A	268565893	10.64	3.15×10^{-5}	0.05	4.50	0.02
7D	1240012	1240012|F|0-23:C>T-23:C>T	150762254	10.63	3.19×10^{-5}	0.05	4.50	1.11
