# Supplementary material for: Genome-Wide Association Study for Spot Blotch Resistance in Synthetic Hexaploid Wheat
Source: Genes (Basel). 2022 Aug 4;13(8):1387. doi: 10.3390/genes13081387 (PMC9407756; doi:10.3390/genes13081387)
Supplement: Supplementary file 1 [file genes-13-01387-s001.zip › Supplementary Table S3.pdf]

Table S3. Significant markers associated with seedling resistance to spot blotch when a DArTSeq consensus genetic map was used**.** Chromosome (Chr), Marker ID**,** Allele ID, genetic position in cM, F statistics, Probability (Prob), Marker R^2, $-\log_{10}$ (*p*-value) and the effect of allele substitution are given for each marker.

Chr.	Marker ID	Allelel ID	Genetic position on consensus map (cM)	F statistic	Prob	Marker R^2	−log10 *p*-value	Effect of allele substitution (genotype effect)
1B	1145134	1145134|F|0-37:T>C-37:T>C	98.03	11.37	1.58×10^{-5}	0.06	4.80	-0.05
1B	1698662	1698662|F|0-37:G>C-37:G>C	148.15	9.28	1.15×10^{-5}	0.05	3.94	-0.26
1B	5582520	5582520|F|0-11:G>A-11:G>A	96.91	8.39	2.70×10^{-4}	0.04	3.57	-0.26
1B	7335825	7335825|F|0-10:C>T-10:C>T	52.56	7.76	4.96×10^{-4}	0.04	3.30	-0.19
1B	1125496	1125496|F|0-23:T>C-23:T>C	51.29	12.12	5.53×10^{-4}	0.03	3.26	NaN
1B	100033209	100033209|F|0-6:A>G-6:A>G	139.32	7.22	8.35×10^{-4}	0.04	3.08	-0.66
1B	4261287	4261287|F|0-17:C>T-17:C>T	51.29	7.05	9.83×10^{-4}	0.04	3.01	-0.29
1D	1065667	1065667|F|0-21:A>T-21:A>T	12.27	7.86	4.50×10^{-4}	0.04	3.35	0.23
1D	12779374	12779374|F|0-30:G>A-30:G>A	130.64	7.52	6.25×10^{-4}	0.04	3.20	0.00
2A	5573285	5573285|F|0-21:A>G-21:A>G	45.45	7.61	5.74×10^{-4}	0.04	3.24	0.17
2A	3533784	3533784|F|0-39:C>T-39:C>T	123.66	7.06	9.75×10^{-4}	0.04	3.01	-0.13
2B	2243785	2243785|F|0-27:T>C-27:T>C	40.74	8.42	2.62×10^{-4}	0.04	3.58	-0.17
2B	7492146	7492146|F|0-17:G>C-17:G>C	107.03	8.28	3.01×10^{-4}	0.04	3.52	0.24
2B	100031252	100031252|F|0-29:T>C-29:T>C	55.48	14.46	1.66×10^{-4}	0.04	3.78	NaN
2D	2245411	2245411|F|0-21:C>A-21:C>A	118.19	7.08	9.54×10^{-4}	0.04	3.02	-0.14
2D	1122278	1122278|F|0-8:C>A-8:C>A	20.85	7.05	9.80×10^{-4}	0.04	3.01	-0.14
3B	1283998	1283998|F|0-27:G>A-27:G>A	68.53	10.66	3.10×10^{-5}	0.05	4.51	-0.02
3B	4989766	4989766|F|0-16:C>T-16:C>T	19.56	8.72	1.97×10^{-4}	0.04	3.71	0.53
3D	1011260	1011260|F|0-43:A>T-43:A>T	82.16	8.82	1.79×10^{-4}	0.04	3.75	-0.05
3D	1074984	1074984|F|0-15:T>G-15:T>G	61.81	7.10	9.36×10^{-4}	0.04	3.03	-0.16
4A	100036641	100036641|F|0-6:C>A-6:C>A	96.36	12.04	8.42×10^{-6}	0.06	5.07	-0.39
4A	100039440	100039440|F|0-27:G>A-27:G>A	113.91	7.14	8.99×10^{-4}	0.04	3.05	-0.32
4A	1162615	1162615|F|0-50:C>T-50:C>T	96.08	7.08	9.57×10^{-4}	0.04	3.02	-0.26

4D	3023637	3023637|F|0-12:C>T-12:C>T	66.12	7.78	4.86×10^{-4}	0.04	3.31	-0.02
5A	3570010	3570010|F|0-29:G>A-29:G>A	36.99	13.96	2.14×10^{-4}	0.04	3.67	NaN
5A	1086529	1086529|F|0-68:G>T-68:G>T	36.99	8.54	2.35×10^{-4}	0.04	3.63	0.20
5B	1240012	1240012|F|0-23:C>T-23:C>T	98.36	10.62	3.20×10^{-5}	0.05	4.49	1.12
6B	1019955	1019955|F|0-55:A>G-55:A>G	46.69	7.17	8.70×10^{-4}	0.04	3.06	-0.46
7A	990293	990293|F|0-7:G>A-7:G>A	88.42	10.76	2.82×10^{-5}	0.05	4.55	-0.05
7A	1095642	1095642|F|0-36:C>T-36:C>T	75.85	10.73	2.90×10^{-5}	0.05	4.54	-0.29
7A	4002611	4002611|F|0-59:C>G-59:C>G	7.25	9.50	9.34×10^{-5}	0.05	4.03	-0.03
7B	100011110	100011110|F|0-15:G>C-15:G>C	46.26	10.10	5.27×10^{-5}	0.05	4.28	-0.23
