# Supplementary material for: Genome-Wide Association Study for Spot Blotch Resistance in Synthetic Hexaploid Wheat
Source: Genes (Basel). 2022 Aug 4;13(8):1387. doi: 10.3390/genes13081387 (PMC9407756; doi:10.3390/genes13081387)
Supplement: Supplementary file 1 [file genes-13-01387-s001.zip › Supplementary Table S4.pdf]

Table S4. Significant markers associated with seedling resistance to spot blotch based on durum wheat (cv. Svevo) and *Ae. tauschii* reference genomes. Chromosome (Chr.), Marker ID, allele ID, physical positions, F-statistics, Probability (Prob), Marker R^2, $-\log_{10}$ (p-value) and the effect of allele substitution are given for each marker

Chr.	Marker ID	Allele ID	Position	F statistic	Prob	Marker R^2	$-\log_{10}$ (p-value)	Effect of allele substitution (genotype effect)
1B	1145134	1145134|F|0-37:T>C-37:T>C	399260869	11.85	9.99×10^{-6}	0.06	5.00	-0.06
2A	1144884	1144884|F|0-29:C>T-29:C>T	576091993	13.30	2.56×10^{-6}	0.07	5.59	-0.00
2B	1240012	1240012|F|0-23:C>T-23:C>T	196456610	10.85	2.58×10^{-5}	0.05	4.59	1.10
2D	1089634	1089634|F|0-38:A>C-38:A>C	507788062	10.91	2.43×10^{-5}	0.05	4.61	0.04
3A	1074984	1074984|F|0-15:T>G-15:T>G	524698865	7.13	9.10×10^{-4}	0.04	3.04	0.17
3A	2279238	2279238|F|0-47:C>T-47:C>T	477190304	10.33	4.25×10^{-5}	0.05	4.37	0.34
3B	1283998	1283998|F|0-27:G>A-27:G>A	593903783	10.85	2.59×10^{-5}	0.05	4.58	0.01
3B	4992362	4992362|F|0-58:C>T-58:C>T	775474348	7.04	9.91×10^{-4}	0.04	3.00	0.02
4D	2243087	2243087|F|0-35:G>A-35:G>A	54178331	10.84	2.61×10^{-5}	0.05	4.58	0.01
7A	4002611	4002611|F|0-59:C>G-59:C>G	6228579	9.93	6.22×10^{-5}	0.05	4.21	-0.01
