# Supplementary material for: Genome-Wide Association Study for Spot Blotch Resistance in Synthetic Hexaploid Wheat
Source: Genes (Basel). 2022 Aug 4;13(8):1387. doi: 10.3390/genes13081387 (PMC9407756; doi:10.3390/genes13081387)
Supplement: Supplementary file 1 [file genes-13-01387-s001.zip › Supplementary Table S5.pdf]

**Table S5**. Candidate genes for significant marker-trait associations identified from *Triticum aestivum* (IWGSC), *Triticum turgidum* (Svevo.v1) and *Aegilops tauschii* (Aet_v4.0) genomes. Data was obtained from Emsembl https://plants.ensembl.org/ (accessed 15/03/2022)

| Chromosome | Marker ID | Gene | Description |
|---|---|---|---|
| 1D | 1125496 | AET1Gv20777500 | n/a |
| 1D | 12779374 | TraesCS1D02G441400 | n/a |
| 1D | 12779374 | AET1Gv21021400 | n/a |
| 1D | 12779374 | TRITD1Bv1G224330 | Lectin receptor kinase |
| 2B | 1240012 | TRITD2Bv1G075350 | U-box domain-containing protein 4 |
| 2D | 1122278 | TraesCS2D02G054200 | n/a |
| 2D | 2243785 | TraesCS2D02G076500 | n/a |
| 2D | 1089634 | AET2Gv20890600 | n/a |
| 3B | 1283998 | TRITD3Bv1G194800 | Disease resistance protein RPM1 G |
| 3B | 4992362 | TraesCS3B02G520000 | n/a |
| 3B | 4992362 | TRITD3Bv1G257410 | Serpin |
| 3D | 1074984 | TraesCS3D02G291900 | n/a |
| 3D | 1074984 | AET3Gv20689000 | n/a |
| 3D | 1011260 | TraesCS3D02G407000 | Peroxidase |
| 3D | 1011260 | AET3Gv20921800 | n/a |
| 4A | 1351280 | TraesCS4A02G355400 | n/a |
| 5A | 100016153 | TraesCS5A02G146400 | Mannan endo-1,4-beta-mannosidase 6 |
| 5A | 100016153 | TRITD5Av1G111170 | Mannan endo-1,4-beta-mannosidase-like protein |
| 5D | 100016153 | AET5Gv20379200 | Mannan endo-1,4-beta-mannosidase 6 |
| 7A | 4002611 | TraesCS7A02G019400 | n/a |
| 7A | 4002611 | TRITD7Av1G003410 | Pectin lyase-like superfamily protein |
| 7D | 22765212 | TraesCS7D02G278500 | Ribosomal protein |
| 7D | 22765212 | AET7Gv20675900 | n/a |
